# Organic-inorganic fertilization regulates carbon sequestration-emission coupling through soil carbon fractions and bacterial community structure in dryland maize fields

**DOI:** 10.3389/fmicb.2026.1901767

**Published:** 2026-09-28

**Authors:** Wenying Liu, Aixia Xu, Lingling Li, Yongjie Zhou, Isaac Osei-Tutu, Junhng Xie, Yanjie Fang

**Affiliations:** 1 State Key Laboratory of Aridland Crop Science, Gansu Agricultural University, Lanzhou, China; 2 College of Agronomy, Gansu Agricultural University, Lanzhou, China; 3 Seed Industry Research Institute of Gansu Provincial University, Lanzhou, China; 4 CSIR-Crops Research Institute, Kumasi, Ghana; 5 Dryland Agriculture Institute, Gansu Academy of Agricultural Sciences, Lanzhou, Gansu, China

**Keywords:** bacterial community, carbon emissions, carbon sequestration, co-occurrence network, dryland maize, organic-inorganic fertilizer combination

## Abstract

**Introduction:**

Carbon sequestration and emission reduction in farmland ecosystems are central to sustainable agricultural development. This study evaluated whether organic-inorganic fertilization could coordinate soil carbon accumulation, respiratory carbon losses, crop productivity, and bacterial community structure in a semiarid dryland maize system.

**Methods:**

Using a long-term fixed-site field experiment established in 2012, we compared five fertilization regimes during 2024 and 2025: no fertilization (NA), chemical fertilizer alone (CF), combined organic and chemical fertilization (SC), organic fertilizer alone (SM), and maize straw alone (MS). We measured maize productivity, soil total carbon and nitrogen, soil organic carbon and labile carbon fractions, soil CO_2_ emissions, and bacterial community composition.

**Results:**

Fertilization increased grain yield and soil carbon and nitrogen pools, although its effects on the growing-season carbon budget differed among treatments. SC produced the highest grain yield, improved carbon emission efficiency, and maintained positive growing-season net ecosystem productivity within the accounting framework used in this study. Compared with NA, SC and CF increased grain yield by 235.4% and 195.3%, respectively. Although SM and MS increased soil carbon and nitrogen pools, neither improved the growing-season balance between crop production and respiratory carbon losses. MS produced the highest total carbon emissions and the most negative net ecosystem productivity. Fertilization primarily altered bacterial community composition through treatment-specific shifts in dominant taxa. SC and CF favored Gemmatimonadota-related groups, whereas MS enriched copiotrophic and substrate-responsive groups, including Proteobacteria and Bacteroidota.

**Discussion:**

These findings indicate that integrated organic-inorganic fertilization may be more effective than high organic-carbon inputs alone for coordinating maize productivity, soil carbon accumulation, and growing-season carbon performance in semiarid dryland maize systems.

## 1 Introduction

Climate change is intensifying, with profound consequences for agroecosystems (Jin et al., 2024; Zhu et al., 2025). Agriculture must therefore reduce greenhouse gas emissions and enhance soil carbon sequestration while maintaining food security and crop productivity (Li et al., 2025; Bai et al., 2026). Dryland farming system provides an important foundation for agricultural production in northern China. However, it has long faced challenges such as insufficient precipitation, poor soil fertility, and low organic matter accumulation (Zhang et al., 2024a). These factors contribute to a fragile soil carbon pool that is highly sensitive to tillage and fertility management practices (Sun and Zhang, 2023; Shi and Fan, 2025). In the rain**-**fed dryland farming region of the western Loess Plateau, limited water and heat resources exacerbate the difficulties of achieving high and stable maize yields (Wang et al., 2022b). Although the adoption of full film double ridge furrow sowing technology has alleviated water and heat constraints to some extent, significantly enhancing maize yields (Li et al., 2026), the long-term overuse of chemical fertilizers has degraded soil fertility, compromised soil quality, and contributed to substantial greenhouse gas emissions (Wang et al., 2024).

Fertilization is a fundamental management practice for enhancing soil fertility and crop productivity and regulating carbon cycling in agroecosystems (Wu et al., 2023; Bai et al., 2026). Although chemical fertilizers alone can rapidly replenish soil nutrients and increase maize yields in the short term (Li et al., 2026), their long-term application often results in soil fertility degradation (Wang et al., 2025b). Given these limitations, organic fertilizers and the combined application of organic and inorganic fertilizers have received considerable attention. However, current findings regarding the ecological effects of organic materials inputs are inconsistent (Li et al., 2025). On the one hand, organic amendments can promote SOC accumulation by increasing exogenous carbon inputs, improving soil aggregate structure, and enhancing soil nutrient retention (Huang et al., 2024). On the other hand, abundance organic substrates may stimulate soil microbial activity, increase soil respiration, and accelerate organic matter mineralization, thereby increasing CO_2_ emissions (Ma et al., 2025). Therefore, an increase in SOC content does not necessarily indicate an improvement in the net carbon balance. When assessing the effects of organic inputs on soil carbon dynamics, both their application rates and biochemical form should be comprehensively considered. In addition, it is still insufficient to evaluate the carbon effect of different fertilization methods only based on a single index such as SOC content or soil respiration. In recent years, comprehensive indicators such as NEP have been increasingly used to determine whether farmland ecosystems are “carbon sinks” or “carbon sources”, because such indicators can comprehensively reflect the carbon input, carbon output and production process of the system.

At the microbial community level, previous studies have primarily focused on fungi. Compared with inorganic fertilizer alone, the combined application of organic and inorganic fertilizers significantly altered fungal community structure. In particular, mineral fertilizer application reduced the relative abundance of saprotrophic fungi by approximately 40% (Wang et al., 2018). Straw incorporation also significantly affected the composition and functional guilds of fungal communities (Yin et al., 2022). However, the response of bacterial community structure to the combined application of organic and inorganic fertilizers, as well as its relationship with changes in soil carbon composition, remains unclear. In addition, previous studies have generally examined SOC, soil respiration and bacterial community structure separately, lacking a systematic analysis of the coupling relationship between the three. Previous studies have shown that fertilization can change the physical and chemical properties of soil, regulate the structure and function of bacterial community, and then affect the carbon sequestration capacity and carbon emission intensity of soil. Different bacterial groups have significant differences in resource utilization strategy, metabolic efficiency and ecological adaptability. Recent studies have further pointed out that bacterial community composition and its network structure may more accurately characterize the changes of soil ecological functions, especially the carbon cycle function, compared with the total bacterial abundance or the commonly used diversity index.

The soil moisture and temperature of rainfed croplands on the western Loess Plateau showed significant intraseasonal and interannual fluctuations. Intermittent rainfall and associated drying-rewetting cycles can regulate crop root activity, labile carbon availability, soil heterotrophic respiration, and microbial resilience to drought (Amarasinghe et al., 2024), whereas plastic film mulching can change the hydrothermal conditions of near surface soil (Wang et al., 2022a). Previous studies based on the same long-term experimental platform on the semi-arid Loess Plateau have shown that fertilization-induced change in soil CO_2_ emissions are closely associated with bacterial community composition and network characteristics (Wang et al., 2022b, 2024). Organic fertilizer application also affects bacterial communities, soil carbon and nitrogen mineralization, crop yield, and N_2_O emissions (Wang et al., 2023; Xie et al., 2024). However, under equivalent nitrogen inputs, comprehensive studies examining respiratory carbon loss, crop carbon balance, labile carbon fractions, and bacterial network characteristics in response to organic carbon amendments of different forms and application rates remain scarce.

To address these research gaps, this study used a long-term field experiment established in 2012 to systematically evaluate the effects of different fertilization systems on SOC fractions, carbon emissions, crop productivity, bacterial community structure, and co-occurrence network characteristics in dryland maize fields. The following core assumptions were put forward: (i) the combined application of organic and inorganic fertilizers achieves a better balance between crop yield and net carbon gain compared to the use of organic fertilizer or maize straw alone; and (ii) bacterial community composition and network characteristics provide greater explanatory power for variations in carbon emissions and the coupling of sequestration and emissions than bacterial abundance or diversity indices. By analyzing the coupling dynamics of carbon sequestration and carbon emissions under different fertilization strategies and the relationship between them and bacterial community structure, this study aims to provide a theoretical basis for high yield, sustainable and low-carbon management of dryland agroecosystems.

## 2 Materials and methods

### 2.1 Experimental site

This study was conducted during 2024–2025 using a long**-**term field experiment established in 2012 in Mazichuan Village, Lijiabao Town, Anding District, Dingxi City, Gansu Province, China (35°28′N, 104°44′E). The site is in the semi-arid region of the Loess Plateau in south-central Gansu Province, a typical rainfed agricultural area without artificial irrigation. The elevation is 1971 m. At the initiation of the long-term experiment in April 2012, soil properties (0–20 cm) were as follows: field capacity 0.256 cm^3^ cm^−3^, wilting point 0.085 cm^3^ cm^−3^, bulk density 1.17 g cm^**–**3^, soil organic matter 8.87 g kg^**–**1^, pH 8.4, total nitrogen 1.0 g kg^**–**1^, available phosphorus 16 mg kg^**–**1^, and available potassium 139 mg kg^**–**1^.

The site has an annual frost-free period of approximately 140 days, average annual sunshine duration of 2,477 h, and mean annual temperature of 6.4 °C. The accumulated temperatures are 2394 °C for ≥ 0 °C and 2239 °C for ≥ 10 °C. The mean annual precipitation was 390.7 mm. Rainfall during the maize growing seasons in 2024 and 2025 was 320.9 mm and 491.1 mm, respectively (Supplementary Figure S1). The light and water conditions in the experimental area permit only one harvest per year. The soil is loessal, with a loose, uniform texture and good water-holding capacity, making the site suitable for evaluating carbon sequestration emission relationships, and bacterial community associations in rain-fed maize system.

### 2.2 Experimental design

The experiment employed a single factor randomized block design, with no fertilizer treatment (NA) as the control. Four fertilized treatments were imposed, all receiving the same total amounts of nitrogen (200 kg N ha^−1^) and phosphorus (150 kg P_2_O_5_ ha^−1^) but differing in fertilization method. The treatments were: chemical fertilizer alone (CF, 200 kg N ha^−1^); a combination of commercial organic fertilizer and chemical fertilizer (SC, 100 kg N ha^−1^ plus 3.03 t ha^−1^ commercial organic fertilizer); commercial organic fertilizer alone (SM, 6.06 t ha^−1^ commercial organic fertilizer); and maize straw alone (MS, 28.5 t ha^−1^ maize straw). The commercial organic fertilizer (Gansu Daxing Agricultural Technology Co, Gansu, China) was produced from well-decomposed cow manure and contained 3.3% N, 1.0% P, 0.7% K, and 31.1% organic matter, with a pH of 8.4. Maize straw from the previous harvest was air dried, cut into ≤ 5 cm, weighed, and applied to the field plots. The maize straw contained 41.2% C, 0.7% N, 0.4% P, 0.5% K, 41.0% Neutral Detergent Fiber, 27.6% Acid Detergent Fiber, and 13.4% Hemicellulose. In total, five treatments (NA, CF, SC, SM, and MS) were implemented, each with three replicates, resulting in 15 experimental plots. Each plot measured 42.9 m^2^. The maize variety “Xianyu 335” was sown in late-April at a density of 52,500 plants ha^−1^ using the full film double ridge furrow sowing and was harvested in mid-October (Supplementary Figure S2).

### 2.3 Sampling and analysis

At maize maturity, grain from each plot was harvested, air dried, threshed, weighed, and converted to yield on a per hectare basis (kg ha^−1^). Soil samples were collected from each plot at depths of 0–20 cm during three key growth stages: sowing, silking, and maturity. Using an S shaped sampling strategy, nine cores were taken from each plot with a 5 cm diameter soil auger, composited, passed through a 2 mm sieve to remove gravel and maize roots, and then stored. Fresh rhizosphere soil collected at the 2024 harvest was stored at −80 °C for microbial community analysis. The remaining soil was air dried for determination of physicochemical properties.

TC and TN concentrations were determined using a TOC analyzer based on high temperature combustion. SOC was estimated through the K_2_Cr_2_O_7_ oxidation method (Nelson and Sommers, 1996). Soil Particulate Organic Carbon (POC) was determined using the sodium hexametaphosphate dispersion method separated using 5 % (mass/volume) sodium hexametaphosphate as a dispersant, and dispersed soil fractions > 53 μm were considered POC. (Cambardella and Elliott, 1992). Easily oxidizable carbon (EOC) was estimated through the KMnO_4_ oxidation method (Mi et al., 2019). Water soluble organic carbon (WSOC) was extracted from the soil using deionized water (1:5 ratio) at 25 ^?^C and quantified through the K_2_Cr_2_O_7_ oxidation method (Li et al., 2016).

Soil carbon and nitrogen stocks in the 0–20 cm layer were calculated from the measured concentrations and treatment-specific soil bulk density as follows:


$$\begin{array}{l} Stock=C\times BD\times h\times 10,000 \end{array}$$

Where Stock is the soil carbon or nitrogen stock (kg ha^−1^), C is the measured concentration (g kg^−1^), BD is soil bulk density (g cm^−3^), and h is soil depth (0.20 m). Treatment-specific bulk densities were 1.261, 1.2005, 1.1889, 1.1478, and 1.1432 g cm^−3^ for NA, CF, SC, SM, and MS, respectively.

Total soil respiration rate was measured at fixed points using a LI-8100A Automated Soil CO_2_ Flux System (LI-COR Inc., Lincoln, NE, USA). Autotrophic respiration and soil heterotrophic respiration were partitioned using the root separation method. For heterotrophic respiration measurements, a 1.1 m × 1.1 m root exclusion area was established in each plot at maize sowing. A trench 10 cm wide and 70 cm deep was excavated along the inner perimeter of the reserved area, and 400 mesh nylon mesh was installed along the trench before backfilling the soil. This mesh allowed the exchange of soil water, air, and nutrients while preventing root penetration. One respiration collar was installed in the center of each root exclusion area, and heterotrophic respiration was measured using the same instrument, frequency, and schedule as total soil respiration. Based on the soil respiration rate and measurement frequency, total carbon emissions during the maize growing season (TCE, kg C ha^−1^) were calculated as follows (Yu et al., 2012):


$$\begin{array}{l} TCE=\sum \left[\frac{R_{i+1}+R_{i}}{2}\times \left(t_{i+1}-t_{i}\right)\times 0.1584\times 24\right]\times 0.2727\times 10 \end{array}$$

where R represents the soil respiration rate (μmol m^−2^ s^−1^), i+1 and i represent consecutive sampling times, and t is days after sowing. The factors 0.1584 and 0.2727 convert μmol CO_2_ m^−2^ s^−1^ to g CO_2_ m^−2^ h^−1^ and g C m^−2^ h^−1^, respectively. The constants 24 and 10 convert the units of carbon emission to kg C ha^−1^.

Carbon emission efficiency (CEE, kg kg^−1^), defined as maize grain yield per unit carbon emission, was calculated as follows (Qin et al., 2013):


$$\begin{array}{l} CEE=\frac{GY}{TCE} \end{array}$$

where GY represents maize kernel yield (kg ha^−1^), and TCE denotes the total carbon emission during the maize growing season (kg C ha^−1^).

Net ecosystem productivity (NEP, kg C ha^−1^), used to evaluate the cropland ecosystem carbon budget, was calculated as follows (Zhang et al., 2024b):


$$\begin{array}{l} NEP=NPP+Ra-Rs \end{array}$$

$$\begin{array}{l} Ra=Rs-Rh \end{array}$$

where Rs, Ra, and Rh represent total respiration, autotrophic respiration, and heterotrophic respiration, respectively (kg C ha^−1^), and NPP represents the carbon contained in maize biomass produced per unit area. External organic carbon provided in the form of corn straw or commercial organic fertilizer is not included in the net ecosystem productivity (NEP) equation, allowing for the calculation of a consistent growth season balance between net primary productivity (NPP) from crop sources and measured carbon losses through respiration. The effectiveness of different fertilization treatments in coordinating crop carbon gains and carbon losses through respiration can be directly compared under this common accounting boundary.

### 2.4 Microbial sequencing and bioinformatics

To characterize the rhizosphere bacterial community under different fertilization regimes, fresh rhizosphere soil samples collected at the 2024 harvest were subjected to 16S rRNA high throughput sequencing. Total DNA was extracted using the MagPure Soil DNA LQ Kit. DNA concentration, purity, and integrity were assessed using a NanoDrop 2,000 spectrophotometer and agarose gel electrophoresis. Qualified samples were stored at −20 °C. Genomic DNA was used as the template to amplify the V3–V4 region of the bacterial 16S rRNA gene with barcoded primers 343F (5′-TACGGRAGGCAGCAG-3′) and 798R (5′-AGGGTATCTAATCCT-3′) (Nossa et al., 2010) and Takara Ex Taq high-fidelity polymerase. Amplification products were sent to Shanghai Ouyi Biotechnology for library construction and high-throughput sequencing.

For the raw FASTQ data, primers were removed using Cutadapt, followed by quality control with DADA2 (Callahan et al., 2016) to generate amplicon sequence variants (ASVs) and their abundance tables (Bolyen et al., 2019). QIIME 2 was used to select representative ASV sequences. Taxonomic assignment was performed against the Silva database (v138) using the q2-feature-classifier plugin. Alpha and beta diversity analyses were conducted in QIIME 2, and inter-group differences were assessed using R packages and ANOVA. Potential bacterial functional profiles were inferred from the representative ASV sequences and abundance table using PICRUSt2 version 2.3.0b0 (Douglas et al., 2020).

### 2.5 Statistical analysis

Data was organized in Excel 2019. Statistical analyses and plotting were conducted using SPSS 29.0, R 4.4.3, Origin 2024, whereas PLS path modeling was conducted using SmartPLS 4. The measured variables were subjected to two-way ANOVA to assess the effects of treatment, year, and their interaction. Mean separation was performed using Duncan's multiple range test at *p* < 0.05. Temporal dynamics of soil carbon and nitrogen components were analyzed with respect to treatment, sampling period, and soil layer. Pearson correlation analysis was used to examine relationships between soil carbon/nitrogen indices and bacterial characteristics.

A random forest model was constructed using the randomForest package in R to identify key factors influencing TCE. The model was run with 1,000 regression trees (ntree = 1,000) with the random seed (set.seed = 123). Variable importance was evaluated using the percentage increase in mean squared error (%IncMSE). The significance of variable importance was assessed using a permutation test with 100 permutations. Model performance was evaluated using the out of bag (OOB) predictions from which the coefficient of determination (*R*^2^) and root mean square error (RMSE) and mean absolute error were also calculated (MAE). Mantel tests and correlation heatmaps were generated with the linkET package in R. The significance of Mantel correlations was assessed using 999 permutations. Correlation strength was classified according to Mantel's r values (< 0.2, 0.2 – 0.4, and ≥ 0.4), and significance levels were classified as *p* < 0.01, *p* < 0.05, and *p* ≥ 0.05.

Before co-occurrence network construction, low abundance and low frequency ASVs were removed to reduce spurious correlations. The ASV abundance table was first converted to relative abundance for each sample, and ASVs with a relative abundance greater than 0.02% in at least 20% of the samples were retained. The bacterial co-occurrence network was constructed at the ASV level using the Hmisc and igraph packages in R. Spearman correlation coefficients were calculated among ASVs using the rcorr() function. Only strong and significant correlations were retained (|r| ≥ 0.6 and Benjamini Hochberg adjusted *p* < 0.01). The filtered correlation matrix was converted into an undirected weighted network, and self-correlations, duplicated edges, and isolated nodes were removed. Network level and node level topological parameters and module structure were calculated using the igraph package. The Gephi software (v0.9.2) was used to construct the co-occurrence network. The network modules were defined as groups of densely interconnected ASVs within the co-occurrence network (Layeghifard et al., 2017). The first principal component of each module (module eigengenes) was used to represent the data of the co-occurrence network. ASVs with degree > 5, closeness centrality > 0.30 and betweenness centrality < 1,000 were retained. This screening procedure identified 20 potential topological keystone ASVs, which were subsequently ranked in descending order of degree for presentation (Barberán et al., 2012; Banerjee et al., 2018a; Wang et al., 2024).

A partial least squares path model was constructed using SmartPLS 4 to evaluate potential relationships among soil properties, bacterial community composition, network topological characteristics, and TCE. Path significance was assessed using nonparametric bootstrapping with 10,000 subsamples, two tailed tests, and 95% percentile confidence intervals. Collinearity was evaluated using inner model variance inflation factors. Model explanatory power and effect sizes were assessed using *R*^2^ and *f* ^2^, respectively, and approximate model fit was evaluated using SRMR and NFI. Because all constructs were represented by single indicators, Cronbach's alpha, composite reliability, and average variance extracted were not considered applicable.

## 3 Results

### 3.1 Yield and carbon budget

Fertilization treatments significantly affected maize grain yield (GY), total soil CO_2_ emissions (TCE), carbon emission efficiency (CEE), net ecosystem productivity (NEP), TN, TC, and SOC (all *p* < 0.05; Table 1). Across the two years, SC and CF significantly increased GY by 235.4% and 195.3%, respectively relative to NA, whereas SM and MS produced comparatively smaller increases. For TCE, MS consistently produced the highest values in both years, increasing by 172.6% and 146.2% relative to NA in 2024 and 2025, respectively. The other fertilized treatments increased TCE by 16.1%−56.7% compared with NA. For CEE, SC consistently achieved the highest values, reaching 1.37 in 2024 and 1.41 in 2025, followed by CF; both were significantly higher than the remaining treatments. Relative to NA, CEE increased by 114.9%−184.6% under SC and CF. Within the NEP accounting framework used in this study, SC and CF maintained positive growing season NEP within the accounting boundary used in this study, with increases of 177.1%−265.4% relative to NA. By contrast, NA, SM, and MS showed negative NEP values, with MS showing the lowest NEP.

**Table 1:** Effects of different fertilization treatments on maize grain yield, total carbon emissions, and carbon-related efficiency indices in 2024 and 2025.

Years	Treatment	GY (kg ha^−1^)	TCE (kg C ha^−1^)	CEE (kg kg^−1^)	NEP (kg C ha^−1^)	TN (kg N ha^−1^)	TC (kg C ha^−1^)	SOC (kg C ha^−1^)
2024	NA	3330.66 ± 223.73 d	6734.38 ± 462.50 c	0.5 ± 0.07 c	−2383.81 ± 499.85 b	2691.96 ± 17.73 c	54979.47 ± 371.51 b	17578.34 ± 335.85 b
	CF	9162.23 ± 1247.64 ab	8471.36 ± 605.56 c	1.08 ± 0.12 b	1988.14 ± 480.84 a	2727.82 ± 31.33 bc	53377.16 ± 666.87 b	18231.59 ± 675.89 b
	SC	10705.17 ± 133.47 a	7817.09 ± 157.03 c	1.37 ± 0.02 a	3298.38 ± 263.91 a	2812.18 ± 49.77 b	55605.82 ± 602.51 b	19482.11 ± 475.63 ab
	SM	4976.95 ± 517.16 cd	10552.96 ± 649.91 b	0.48 ± 0.07 c	−4331.08 ± 842.22 b	2836.58 ± 27.39 b	56403.4 ± 895.45 b	18334.19 ± 937.21 b
	MS	6980.21 ± 828.82 bc	18360.25 ± 852.40 a	0.38 ± 0.05 c	−8778.20 ± 1186.03 c	3204.12 ± 31.18 a	61201.42 ± 1603.95 a	20791 ± 590.69 a
2025	NA	4044.81 ± 312.03 c	8151.50 ± 220.27 c	0.49 ± 0.04 bc	−3707.90 ± 54.19 b	2345.46 ± 25.22 d	52020.45 ± 189.48 c	17306.72 ± 464.22 c
	CF	12616.32 ± 1643.11 a	9813.06 ± 347.30 b	1.3 ± 0.35 a	2687.66 ± 462.90 a	2777.16 ± 21.17 b	51477.44 ± 504.21 c	18121.8 ± 319.02 c
	SC	14033.06 ± 453.00 a	9959.91 ± 185.37 b	1.41 ± 0.04 a	3721.64 ± 839.45 a	2766.17 ± 34.55 b	53873.02 ± 69.1 b	22431 ± 209.62 ab
	SM	6670.38 ± 156.80 b	9550.40 ± 133.62 bc	0.7 ± 0.04 b	−2032.33 ± 98.32 b	2548.12 ± 13.25 c	55691.26 ± 68.87 a	21421.81 ± 428.44 b
	MS	6120.97 ± 300.71 bc	20072.39 ± 984.68 a	0.31 ± 0.03 c	−13739.65 ± 778.18 c	2896.11 ± 46.36 a	56771.31 ± 563.31 a	22589.51 ± 337.04 a
(T)		*F (4, 20)* = 46.33; *p* < 0.001	*F* (4, 20) = 151.02; *p* < 0.001	*F* (4, 20) = 59.73; *p* < 0.001	*F* (4, 20) = 162.78; *p* < 0.001	*F* (4, 20) = 73.22; *p* < 0.001	*F* (4, 20) = 26.16; *p* < 0.001	*F* (4, 20) = 27.20; *p* < 0.001
(Y)		*F* (1, 20) = 12.18; *p* = 0.002	*F* (1, 20) = 10.69; *p* = 0.004	*F* (1, 20) = 2.20; *p* = 0.154	*F* (1, 20) = 1.95; *p* = 0.178	*F* (1, 20) = 87.44; *p* < 0.001	*F* (1, 20) = 27.89; *p* < 0.001	*F* (1, 20) = 35.45; *p* < 0.001
(T) × (Y)		*F* (4, 20) = 2.91; *p* = 0.048	*F* (4, 20) = 2.56; *p* = 0.070	*F* (4, 20) = 1.29; *p* = 0.307	*F* (4, 20) = 9.12; *p* < 0.001	*F* (4, 20) = 15.61; *p* < 0.001	*F* (4, 20) = 2.02; *p* = 0.131	*F* (4, 20) = 10.25; *p* < 0.001

The abbreviations NA, CF, SC, SM, and MS refer to the following treatments: no fertilization, chemical fertilizer only, 50% chemical fertilizer combined with 50% organic fertilizer, organic fertilizer only, and maize straw only, respectively. Values are presented as means ± SD (*n* = 3). Different lowercase letters indicate significant differences among treatments (*p* < 0.05). GY denotes grain yield; TCE represents total carbon emissions; CEE stands for carbon emission efficiency; NEP indicates net ecosystem productivity. T represents the treatment effect, Y denotes the year effect, and T × Y indicates the interaction effect between treatment and year; Two-way ANOVA results are reported as F (df1, df_2_) and *p*-values.

Among the treatments, MS produced the greatest increases in both TN and TC. Across the two years, TN was highest under MS, averaging 21.1% relative to NA. The corresponding average increases under CF, SC, and SM were 9.3%, 10.7%, and 6.9%, respectively. TC showed a similar pattern, with MS consistently achieving the highest values, corresponding to an average increase of 12.5% relative to NA, whereas CF showed a slight average decrease of 2.0%. The response of SOC to fertilization was less consistent than that of TC and TN. In 2024, SOC was highest under MS and SC, with MS increasing SOC by 18.3% compared to NA. In contrast, in 2025, SOC peaked under SM and SC, both significantly exceeding NA and CF. Overall, organic inputs were more effective than mineral fertilization in promoting the accumulation of soil carbon and nitrogen pools. MS produced the most pronounced improvements in TN and TC, whereas SC and SM conferred greater advantages in enhancing SOC.

### 3.2 Soil respiration dynamics and component partitioning

During the growing season, total soil respiration showed a unimodal pattern, peaking around 90 days after sowing (Figure 1). MS consistently showed the highest total soil respiration rate, reaching 16.80 and 18.85 μmol m^−2^ s^−1^ in 2024 and 2025, respectively, which were 110.0% and 110.2% higher than NA. Accordingly, cumulative soil carbon emissions under MS reached 18,360 and 20,072 kg C ha^−1^, increasing by 172.6% and 146.2% relative to NA in 2024 and 2025, respectively.

**Figure 1:**
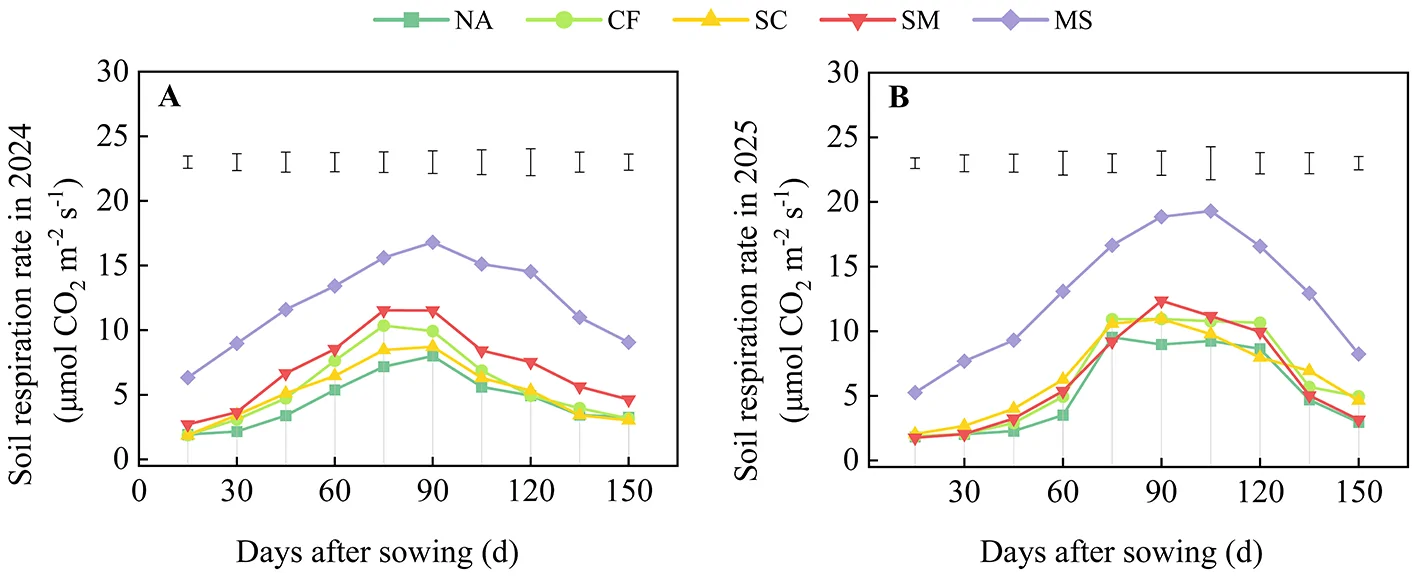
Variation in total soil respiration rate under different fertilization treatments in 2024 and 2025. **(A)** and **(B)** represent the 2024 and 2025 maize growing seasons, respectively. Rs, total soil respiration rate; NA, no fertilizer; CF, chemical fertilizer; SC, combined organic and inorganic fertilizer; SM, organic fertilizer alone; MS, maize straw alone. Error bars indicate standard error.

Autotrophic respiration peaked around 75 days after sowing, with CF and SC reaching the highest rates of 2.71 and 2.94 μmol m^−2^ s^−1^, respectively (Figures 2A, C), increasing by 86.6% and 102.8% compared with NA. Cumulative autotrophic CO_2_ emissions were also highest under CF and SC, increasing by 149.6% and 163.2%, respectively. In contrast, heterotrophic respiration peaked around 90 days after sowing and was highest under MS, with a peak value of 17.16 μmol m^−2^ s^−1^, which was 115.1% higher than NA (Figures 2B, D). Cumulative heterotrophic CO_2_ emissions under MS were 165.9%, 155.9%, 171.8%, and 137.9% higher than those under NA, CF, SC, and SM, respectively.

Regarding the contributions of soil respiration components (Figure 2E), autotrophic respiration contributed the most under CF and SC, accounting for 26% and 28%, respectively. Heterotrophic respiration displayed the opposite pattern: MS had the highest contribution (98%), followed by NA. Relative to NA, MS increased the contribution of heterotrophic respiration by 12.6%, whereas CF, SC, and SM showed significant decreases of 14.9%, 17.2%, and 4.6%, respectively.

**Figure 2:**
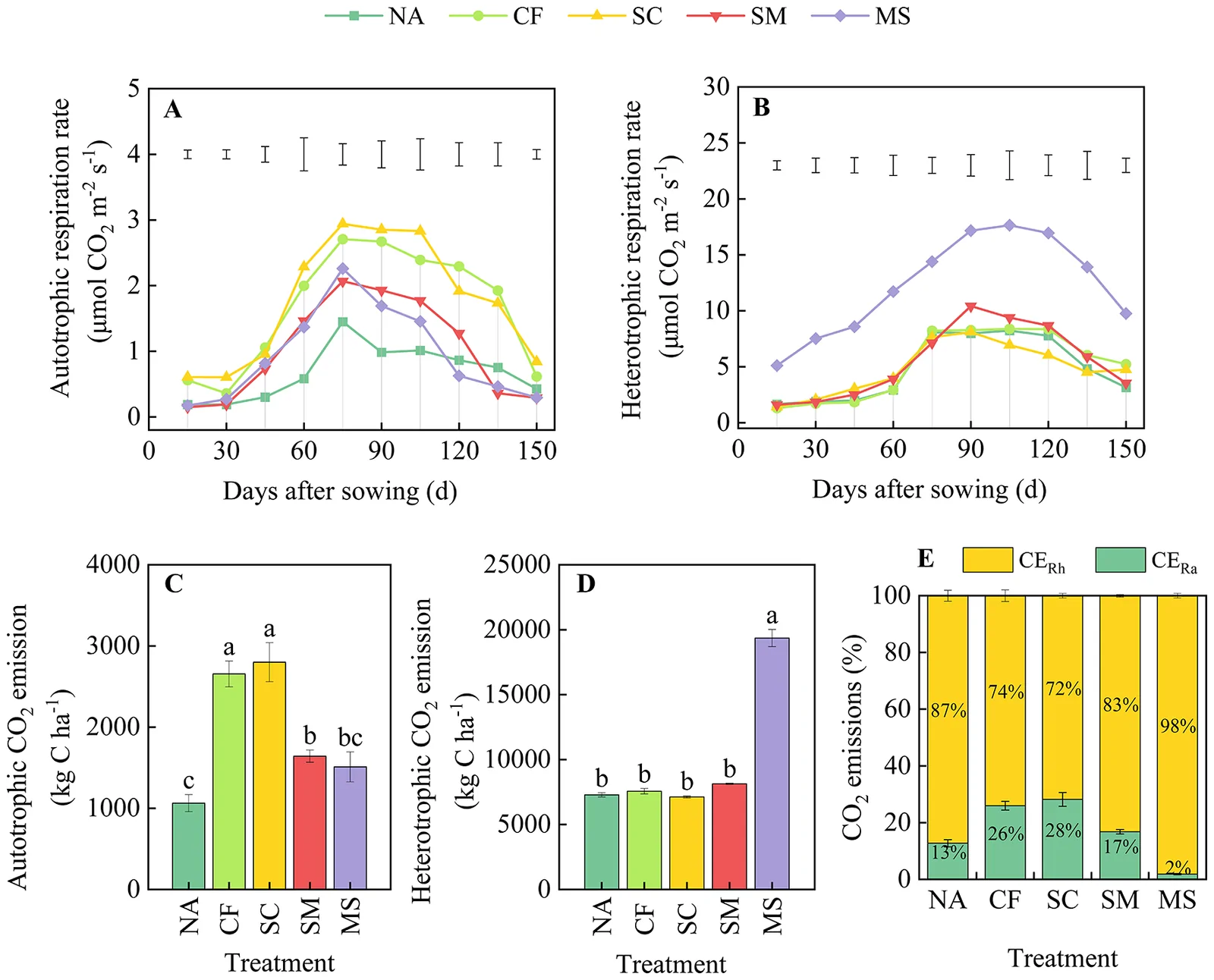
Relative contributions of soil respiration components under different fertilization regimes. **(A)** dynamics of autotrophic respiration; **(B)** dynamics of heterotrophic respiration; **(C)** cumulative autotrophic CO_2_ emissions; **(D)** cumulative heterotrophic CO_2_ emissions; **(E)** relative contributions of autotrophic and heterotrophic respiration to total carbon emissions. Ra and Rh represent the autotrophic and heterotrophic respiration rates, respectively. CE_Rh_ and CE_Ra_ denote carbon emissions from heterotrophic and autotrophic respiration, respectively. The abbreviations NA, CF, SC, SM, and MS refer to the following treatments: no fertilization, chemical fertilizer only, 50% chemical fertilizer combined with 50% organic fertilizer, organic fertilizer only, and maize straw only, respectively. Different lowercase letters indicate significant differences among treatments within the same growth stage according to Duncan's multiple range test at *p* < 0.05.

### 3.3 Soil carbon and nitrogen pools and labile carbon fractions

Fertilization regimes significantly influenced TC, TN and SOC, with distinct variations among growth stages (*p* < 0.05, Figure 3). TC was consistently highest under MS at the sowing, silking, and maturity stages, with increases of 10.6%, 9.5%, and 9.1% relative to NA, followed by SM. TN was highest under MS and lowest under NA across all growth stages. At sowing, both SC and MS significantly increased TN, by 26.6% and 26.7% compared with NA, respectively. During the silking and maturity stages, TN under all fertilization treatments increased by 8.0%−22.1% and 8.6%−23.5%, respectively, relative to NA. SOC was highest under MS and SM, increasing by 29.1% and 23.1% relative to NA, respectively. During the silking and maturity stages, SOC remained highest under MS, with increases of 41.5% and 30.5% compared to NA. Temporally, TC and TN generally peaked at silking, whereas SOC declined by 6.4%−17.5% over the growing season, with smaller reductions under CF and SC. Overall, MS showed the strongest enhancement of soil carbon and nitrogen pools, while CF had a weaker effect on soil carbon accumulation.

**Figure 3:**
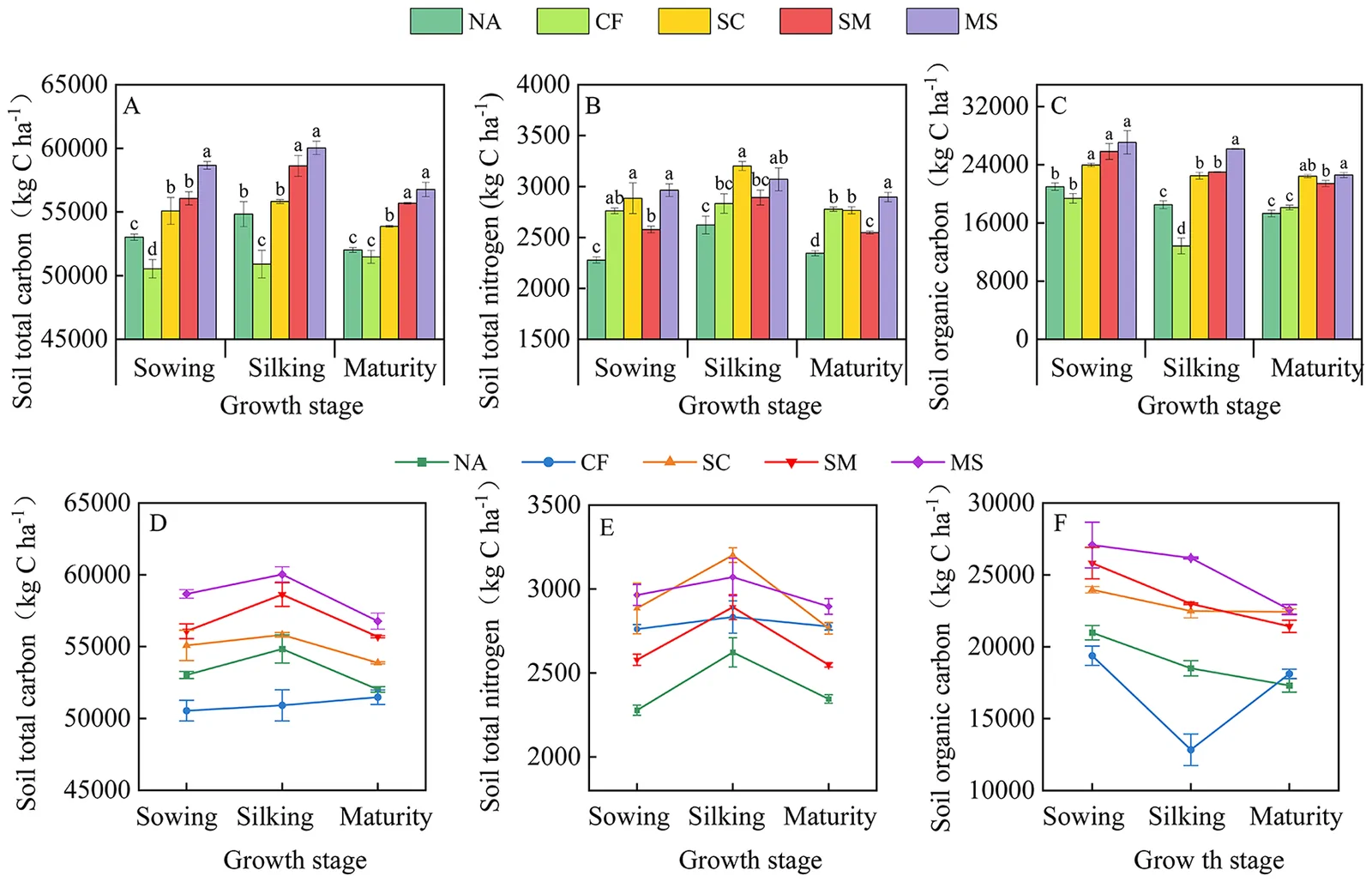
Differences among treatments in total carbon, total nitrogen and SOC at the maize sowing, silking, and maturity stages. **(A–C)** show soil TC, TN, and SOC stocks at different growth stages; **(D–F)** show the seasonal trends of TC, TN, and SOC under different fertilization treatments. TC, TN, and SOC denote soil total carbon, soil total nitrogen, and SOC, respectively. NA, CF, SC, SM, and MS represent no fertilization, chemical fertilizer only, 50% chemical fertilizer combined with 50% organic fertilizer, organic fertilizer only, and maize straw only, respectively. Different lowercase letters indicate significant differences among treatments within the same growth stage according to Duncan's multiple range test at *p* < 0.05.

Fertilization regimes significantly altered labile carbon fractions, including POC, EOC, and WSOC (*p* < 0.05, Figure 4). POC was more responsive to fertilization than SOC and consistently reached its highest levels under MS at all growth stages. At sowing, CF, SC, SM, and MS increased POC by 22.6%, 47.7%, 36.0%, and 65.9%, respectively, relative to NA. During the silking and maturity stages, POC generally followed the order MS > SC > SM > CF > NA, with MS increasing POC by 149.5% and 150.9% over NA, respectively. EOC was also highest under MS at sowing, silking, and maturity, which were 212.9%, 135.9%, and 156.5% higher than NA. WSOC showed the strongest response to organic inputs, with MS reaching 61.9, 81.0, and 78.4 kg ha^−1^ at the three stages, increasing by 346.9%, 237.9%, and 333.6% relative to NA, respectively. Temporally, POC peaked at silking under SC and MS, EOC generally declined during the growing season, whereas WSOC increased from sowing to silking and remained high under MS and SM. Overall, MS most strongly enhanced labile carbon fractions, and POC, EOC, and WSOC were more sensitive to fertilization than SOC.

**Figure 4:**
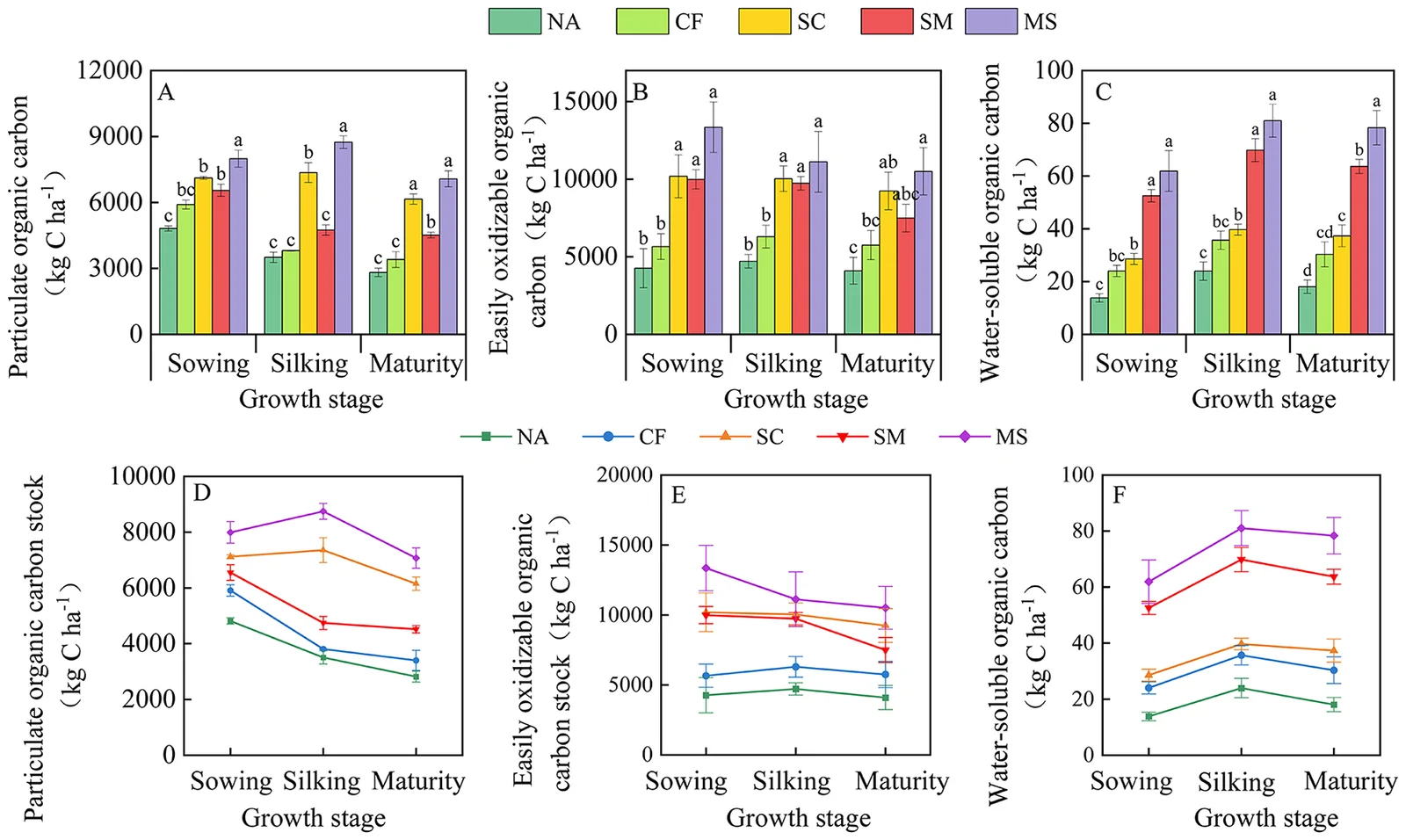
Differences among treatments in POC, EOC and WSOC at the maize sowing, silking, and maturity stages. **(A–C)** show POC, EOC, and WSOC stocks at different growth stages; **(D–F)** show the seasonal trends of POC, EOC, and WSOC under different fertilization treatments. POC, EOC, and WSOC denote particulate organic carbon, easily oxidizable carbon, and water-soluble organic carbon, respectively. NA, CF, SC, SM, and MS represent no fertilization, chemical fertilizer only, 50% chemical fertilizer combined with 50% organic fertilizer, organic fertilizer only, and maize straw only, respectively. Different lowercase letters indicate significant differences among treatments within the same growth stage according to Duncan's multiple range test at *p* < 0.05.

### 3.4 Bacterial community composition and co-occurrence network

Analysis of 16S rRNA sequencing data from rhizosphere soil samples collected at harvest in 2024 showed that different fertilization methods significantly affected the composition and structure of the maize rhizosphere bacterial community (Figure 5). A comprehensive analysis of the 16S rRNA gene sequences identified 35 phyla, 90 classes, 231 orders, 346 families, 607 genera, and 1,083 species.

**Figure 5:**
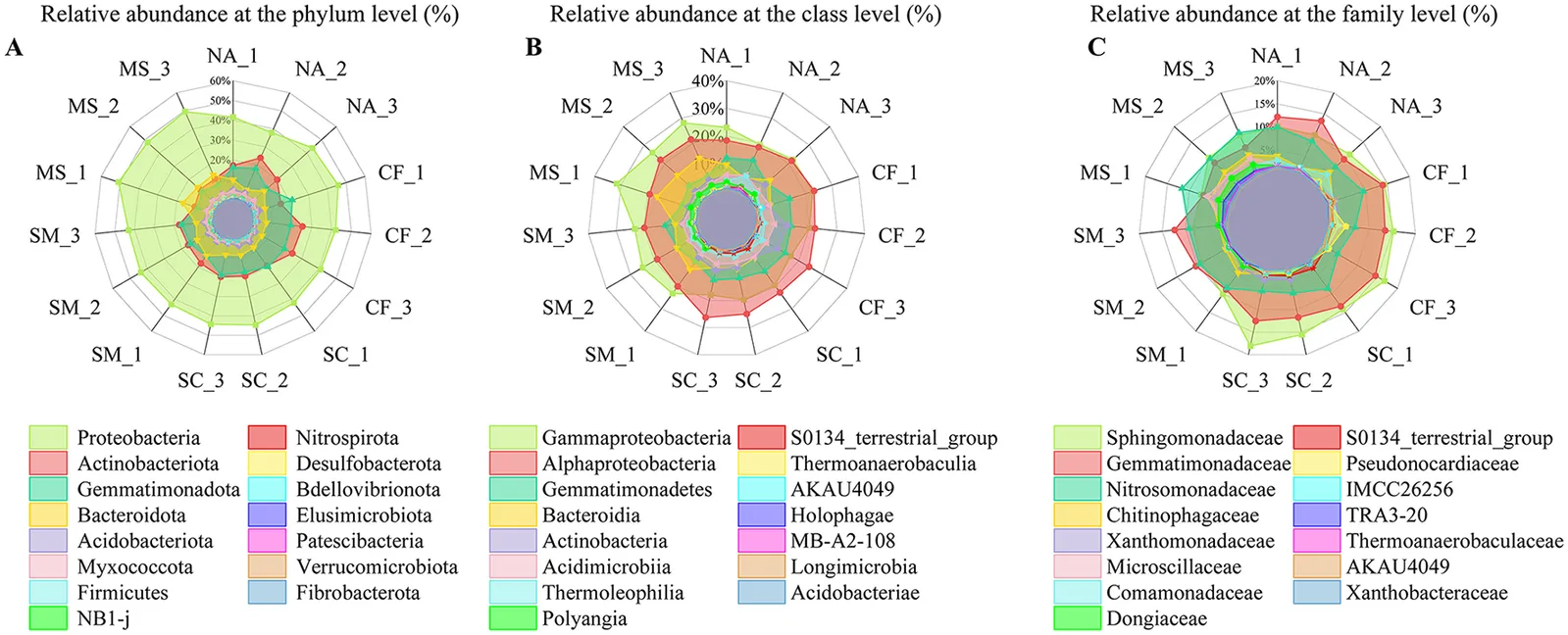
Effects of different fertilization regimes on the relative abundance of soil bacterial communities at the phylum, class, and family levels. **(A)** Phylum level; **(B)** class level; and **(C)** family level. NA, CF, SC, SM, and MS represent no fertilization, chemical fertilizer only, 50% chemical fertilizer combined with 50% organic fertilizer, organic fertilizer only, and maize straw only, respectively.

At the phylum level, the predominant bacterial groups were Proteobacteria (37.9%−50.7%), Actinobacteriota (10.3%−25.1%), Gemmatimonadota (8.7%−21.4%), and Bacteroidota (6.2%−16.6%). Together, these phyla accounted for 73.1%−89.8% of the total relative abundance and constituted the core groups underpinning soil bacterial ecological functions. Among the treatments, CF significantly increased the relative abundances of Actinobacteriota and Gemmatimonadota, while markedly reducing those of Bacteroidota, Acidobacteriota, Myxococcota, Firmicutes, and NB1-j by 23.9%, 30.2%, 55.8%, 46.9%, and 65.5%, respectively, compared with NA. Under SC, the relative abundance of Gemmatimonadota increased by 18.9% compared with NA, whereas the relative abundances of Actinobacteriota, Acidobacteriota, Myxococcota, and Firmicutes decreased by 4.7%, 20.7%, 46.9%, and 35.9%, respectively. SM increased the relative abundances of Bacteroidota and Firmicutes by 18.6% and 108.2%, respectively, compared with NA, while Actinobacteriota decreased by 16.5%. In contrast, MS showed an opposite regulatory pattern to CF, significantly increasing the relative abundances of Proteobacteria, Bacteroidota, and NB1-j by 20.3%, 65.5%, and 28.6%, respectively.

At the class level, the dominant groups were Gammaproteobacteria, Alphaproteobacteria, Gemmatimonadetes, and Bacteroidia. CF and SC significantly increased Alphaproteobacteria but decreased Gammaproteobacteria and Polyangia. Notably, MS resulted in the highest relative abundances of Gammaproteobacteria and Bacteroidia, with increases of 29.1% and 78.2%, respectively, compared with NA. At the family level, the core dominant families were Sphingomonadaceae, Gemmatimonadaceae, and Nitrosomonadaceae. CF and SC significantly increased the relative abundance of Sphingomonadaceae by 43.7% and 48.6%, respectively, compared with NA. MS resulted in the highest relative abundances of Nitrosomonadaceae, Chitinophagaceae, Microscillaceae, and Dongiaceae, with increases of 45.3%, 33.9%, 139.6%, and 57.8%, respectively.

Overall, CF and SC shifted the bacterial community toward greater relative abundances of Actinobacteriota, Gemmatimonadota, and Sphingomonadaceae. In contrast, MS favored Proteobacteria and Bacteroidota and increased the relative abundance of Nitrosomonadaceae. These taxa are commonly associated with rapid responses to available organic substrates, organic matter decomposition, and nitrogen transformation.

After low abundance ASVs were filtered, a bacterial co-occurrence network was constructed from the retained ASVs using Spearman correlations, followed by topological analysis and identification of potential topologic al keystone ASVs (Figure 6). The network comprised 486 nodes and 4,252 edges, with an average degree of 17.50, an average weighted degree of 13.977, an average clustering coefficient of 0.433, an average path length of 4.474, and a modularity index of 0.456. At the phylum level (Figure 6A), Proteobacteria, Gemmatimonadota and Actinobacteriota dominated the network, accounting for 39.30%, 21.19% and 17.28% of the total nodes, respectively. At the class level (Figure 6B), Gammaproteobacteria, Alphaproteobacteria, and Gemmatimonadetes were the major groups, accounting for 23.87%, 15.43%, and 13.99%, respectively, establishing them as the primary constituent clades within the network. At the family level (Figure 6C), Gemmatimonadaceae, Nitrosomonadaceae, and Sphingomonadaceae were the dominant families, accounting for 13.99%, 8.64% and 7.41%, respectively. Module partitioning analysis further revealed a well-defined modular structure within the network (Figure 6D). The network was divided into six major modules, which accounted for 27.37%, 25.10%, 15.64%, 14.61%, 12.14%, and 5.14% of the total nodes, respectively. Nodes within these modules exhibited stronger intra module connections, while inter-module connections were relatively sparse.

**Figure 6:**
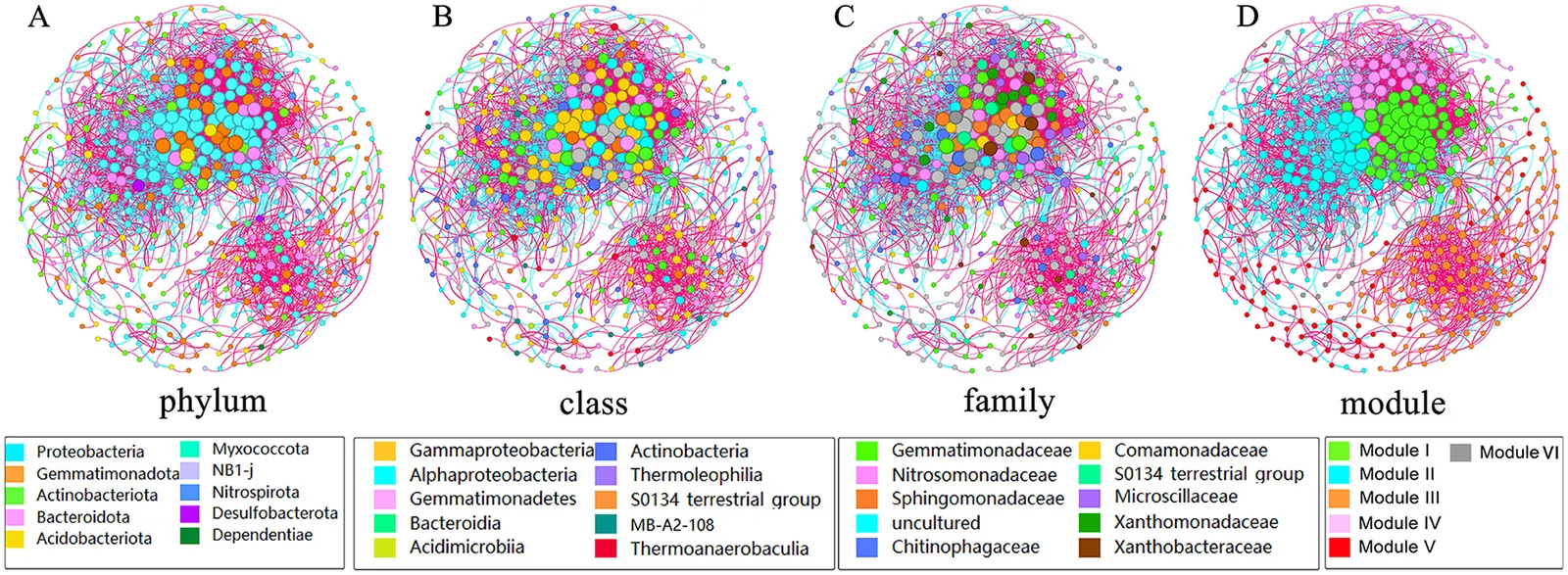
Distribution characteristics of soil bacterial co-occurrence network nodes at the phylum, class, family, and module levels. **(A)** Phylum level; **(B)** class level; **(C)** family level; and **(D)** module level.

Several ASVs demonstrated high connectivity and centrality within the bacterial co-occurrence network and were therefore operationally classified as potential topological keystone ASVs (Supplementary Table S1; Supplementary Figure S3). These ASVs were identified based on the following criteria: degree > 5, betweenness centrality < 1,000, and closeness centrality > 0.30. Applying these thresholds identified 20 ASVs, were identified, predominantly affiliated with the phyla Proteobacteria, Gemmatimonadota, Bacteroidota, and Actinobacteriota. At the class level, they were primarily assigned to Gammaproteobacteria, Alphaproteobacteria, Gemmatimonadetes, Bacteroidia, Longimicrobia, and Actinobacteria. At the family level, they were affiliated with Xanthomonadaceae, Gemmatimonadaceae, Nitrosomonadaceae, Longimicrobiaceae, Sphingomonadaceae, Microscillaceae, Chitinophagaceae, and Xanthobacteraceae.

Among the 20 potential topological keystone ASVs, degree ranged from 43 to 66, betweenness centrality ranged from 176.51 to 917.16, closeness centrality ranged from 0.30 to 0.32, and eigenvector centrality ranged from 0.44 to 0.75. ASV52, affiliated with Xanthomonadaceae, had the highest degree (66), indicating that it was the most highly connected node in the network. ASV7, affiliated with Sphingomonadaceae, exhibited the highest betweenness centrality (917.16), whereas ASV603, affiliated with Xanthobacteraceae, had the highest eigenvector centrality (0.75), indicating strong associations with other highly connected nodes. Most potential topological keystone ASVs were located in Modules I and II, suggesting that they occupied central positions in the inferred network. However, their topological prominence alone is insufficient to establish their biological role as keystone taxa.

### 3.5 PICRUSt2 predicted bacterial functional profiles and their associations with soil carbon fractions

Metabolism was the dominant PICRUSt2 **inferred** KEGG Level 1 category under all treatments. At KEGG Level 2, carbohydrate metabolism, energy metabolism, amino acid metabolism, lipid metabolism, and glycan biosynthesis and metabolism, showed relatively high predicted abundances across treatment, representing potential bacterial functions related to soil carbon transformation. However, the overall profiles under NA, CF, SC, SM, and MS were broadly similar, indicating limited differences in specific predicted categories rather than a pronounced restructuring of the overall inferred KEGG profile (Supplementary Figure S4). Correlation analysis showed that most predicted carbon related KEGG pathways were positively associated with TC, TN, POC, and WSOC (Figure 7). Signal transduction was significantly positively correlated with TC, TN, and POC (*r* = 0.56–0.67, *p* < 0.05), while energy metabolism, glycan biosynthesis and metabolism, transport and catabolism, and metabolism of cofactors and vitamins were significantly correlated with TN and POC (*r* = 0.52–0.60, *p* < 0.05). These results indicate that the inferred functional profiles covaried with soil carbon and nitrogen pools, particularly POC. However, because PICRUSt2 predictions are derived from 16S rRNA marker gene data, these associations do not demonstrate actual gene abundance, pathway expression, enzyme activity, metabolic rates, or causal effects on carbon and nitrogen turnover.

**Figure 7:**
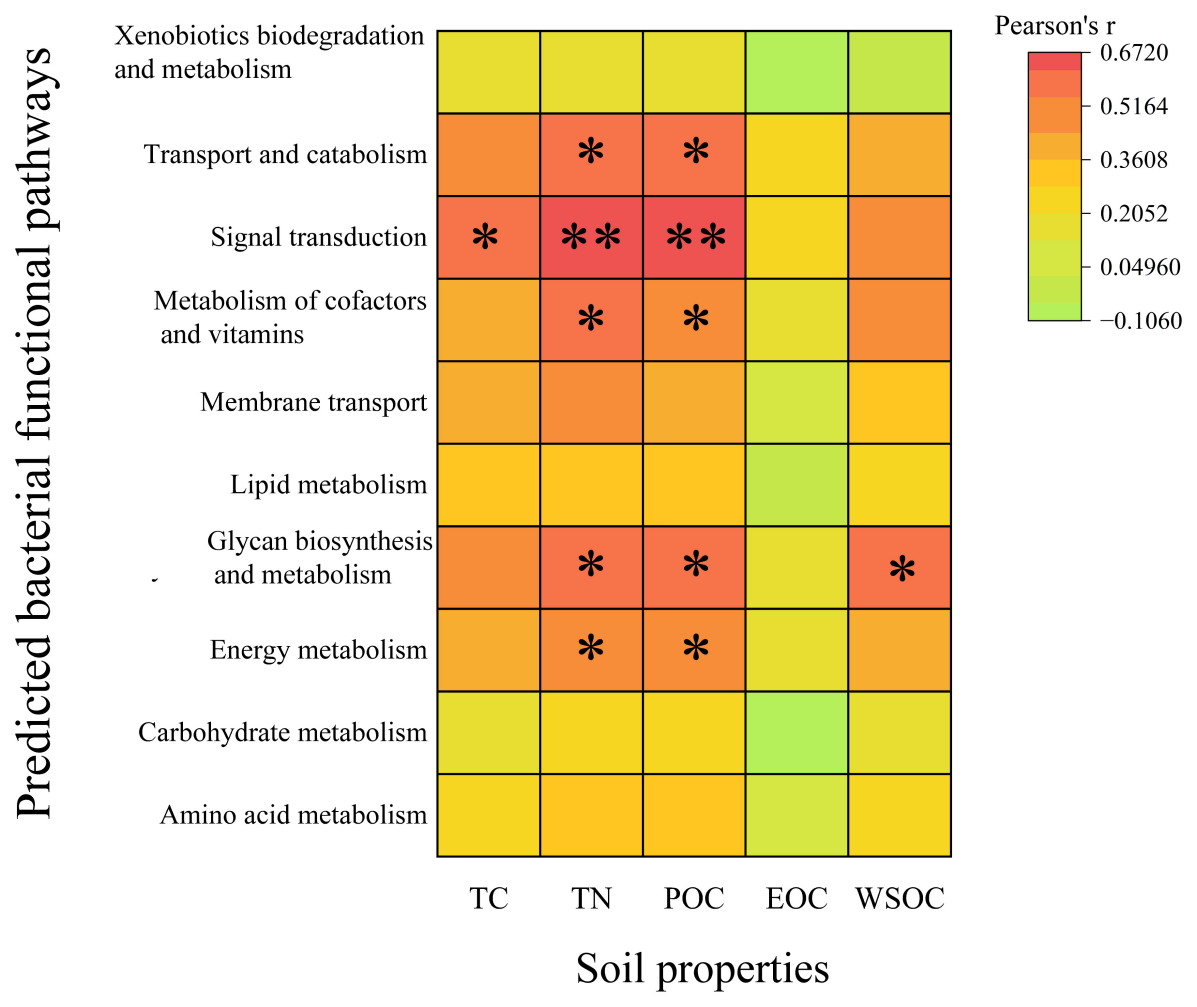
Relationships between predicted bacterial functional pathways and soil properties under different fertilization regimes. While * and ** denote significance at the *p* < 0.05 and *p* < 0.01 levels, respectively.

### 3.6 Soil carbon fractions and bacterial community characteristics jointly explain variation in carbon emission

To clarify the factors contributing to CO_2_ emissions from farmland soils, a random forest model was applied to assess the relative importance of soil properties and bacterial community characteristics (Figure 8). The out of bag (OOB) evaluation yielded *R*^2^ of 0.878 and RMSE of 1522.26, and an MAE of 1,166.07. The model identified the most influential predictors of total carbon emissions based on importance of permutation.

**Figure 8:**
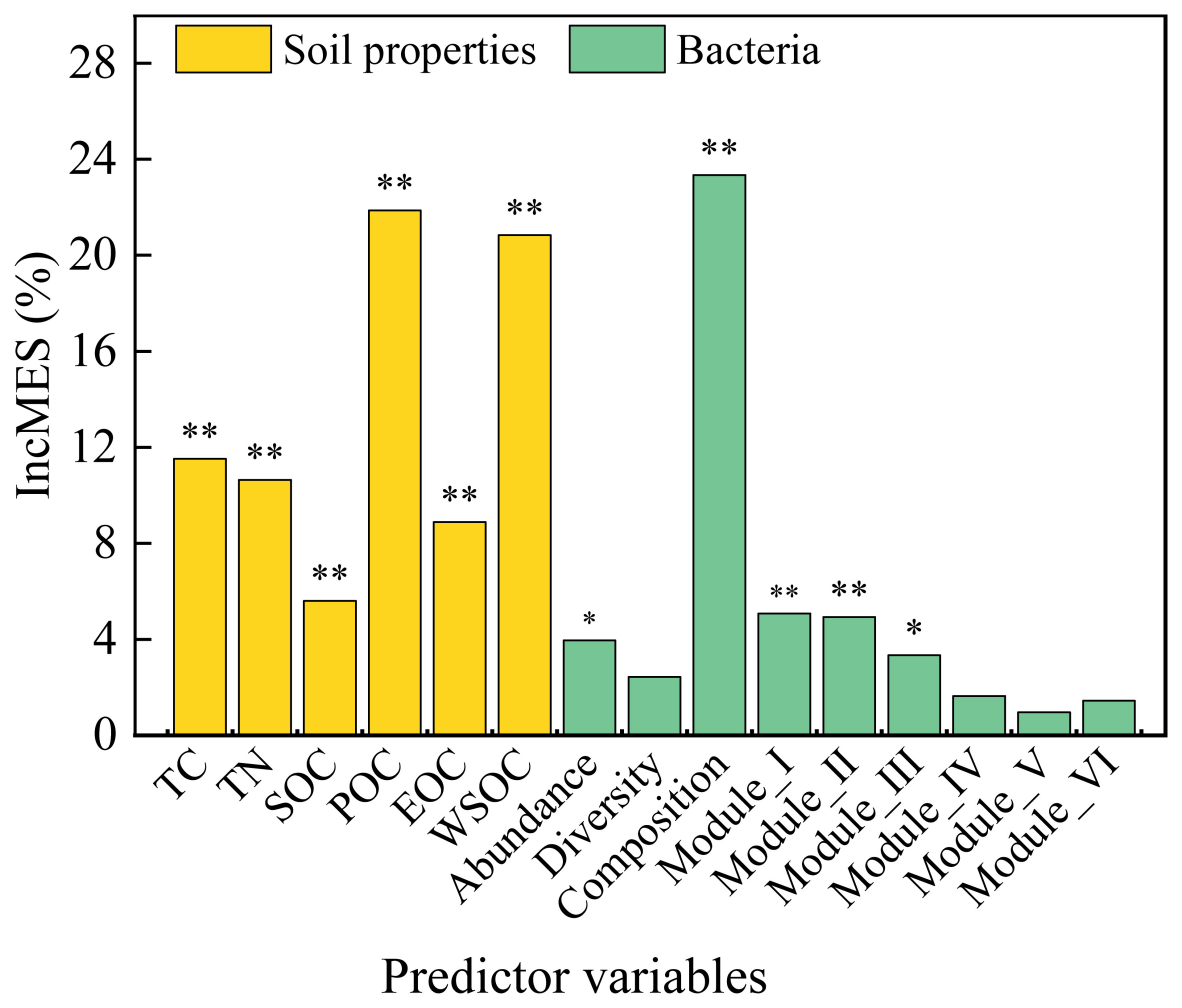
Importance of soil properties and bacterial community characteristics for predicting total carbon emissions in cropland, as determined by the random forest model. IncMSE indicates the percentage increase in mean squared error. ^*^ and ^**^ indicate significance at *p* < 0.05 and *p* < 0.01, respectively.

Among all predictors, bacterial community composition had the highest importance (23.35%). Among soil properties, labile carbon fractions were the main soil properties associated with TCE, with POC and WSOC showing the highest importance values of 21.87% and 20.83%, respectively. TC, TN, EOC and SOC exhibited importance values of 11.52%, 10.65%, 8.89%, and 5.60%, respectively, all at highly significant levels. By contrast, bacterial community diversity and abundance had lower values. For bacterial co-occurrence network modules, Module I and Module II had relatively higher importance values of 5.08% and 4.93%, respectively; overall, TCE was mainly associated with bacterial community composition and labile carbon fractions, especially POC and WSOC.

Linear regression analyses further showed contrasting associations between dominant bacterial taxa and soil carbon and nitrogen indices as well as TCE (Supplementary Figures S5–S7). Proteobacteria, Gammaproteobacteria, and Nitrosomonadaceae were positively associated with TCE, TC, and TN, whereas Gemmatimonadota, Gemmatimonadetes, and Gemmatimonadaceae showed negative associations with these variables.

Pearson correlation analysis, Mantel tests, and multidimensional association analyses indicated substantial relationships among soil carbon components, bacterial community characteristics, and network modules (Figure 9). TC, TN, POC, EOC and WSOC were generally positively correlated with each other, and bacterial community composition was significantly positively correlated with these soil carbon and nitrogen variables. In contrast, Module II was negatively correlated with TC, TN, POC, EOC, and WSOC. The Mantel test further clarified the overall correlation patterns of SOC and TCE with various indicators. SOC showed a highly significant positive correlation with POC (Mantel's *r* = 0.3978, *p* = 0.001), EOC (*r* = 0.2076, *p* = 0.004), WSOC (*r* = 0.3933, *p* = 0.001), bacterial community composition (*r* = 0.1381, *p* = 0.028) and Module II (*r* = 0.3526, *p* = 0.001). TCE exhibited its strongest correlation with POC (*r* = 0.8133, *p* = 0.001), a highly significant positive correlation with bacterial community composition (*r* = 0.7351, *p* = 0.001) and WSOC (*r* = 0.5846, *p* = 0.001). In addition, TCE was significantly or highly significantly positively correlated with TC (*r* = 0.3671, *p* = 0.002), TN (*r* = 0.3564, *p* = 0.006), EOC (*r* = 0.3589, *p* = 0.003), and Module II (*r* = 0.2067, *p* = 0.002).

**Figure 9:**
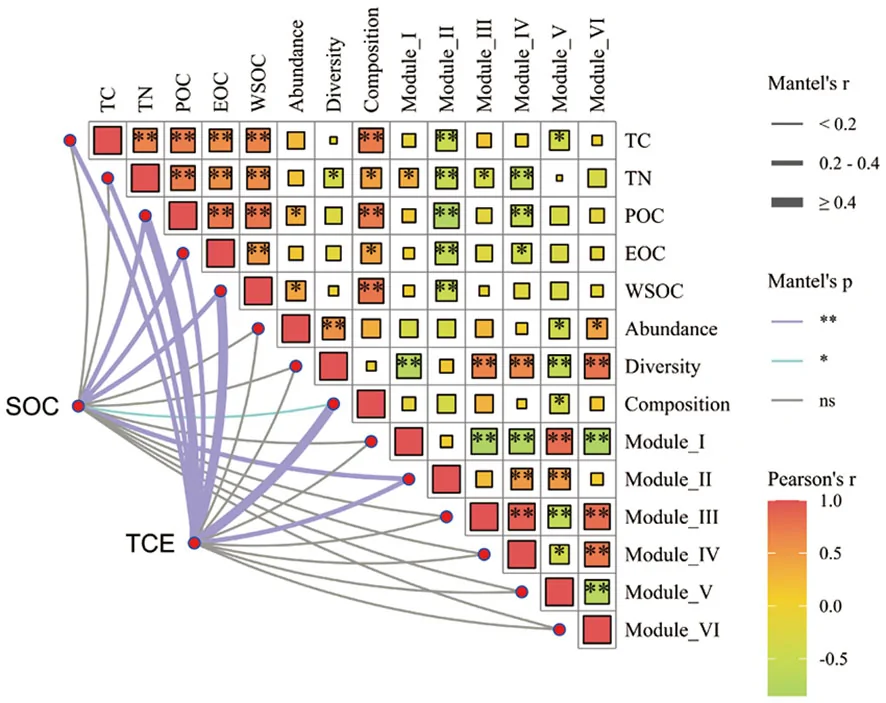
Pearson correlations and Mantel test results among soil carbon fractions, total carbon emissions, and bacterial community attributes. Abundance, Diversity, and Composition represent bacterial community abundance, diversity, and composition, respectively; Modules I–VI represent bacterial co-occurrence network modules. Cell colors indicate Pearson's correlation coefficients, whereas line colors and widths indicate Mantel's p and r values, respectively. * and ** indicate significance at *p* < 0.05 and *p* < 0.01, respectively; ns indicates no significance.

The results of the PLS path model revealed close associations among soil properties, soil bacterial community composition and bacterial network modules and TCE (Figure 10). The model showed acceptable approximate fit, with SRMR = 0.075, Chi-square = 6.036, and NFI = 0.960. Inner model VIF values ranged from 1.000 to 4.129, indicating no severe multicollinearity. The model explained 54.1%, 41.1%, 31.5%, and 92.5% of the variation in bacterial community composition, Module I, Module II, and TCE, respectively. TC and TN were directly associated with TCE, with path coefficients of 0.318 and 0.425, respectively. Both TC and TN also affected TCE through bacterial communities and network modules. Overall, variation in TCE was mainly closely associated with soil properties, bacterial community composition, and network modules.

**Figure 10:**
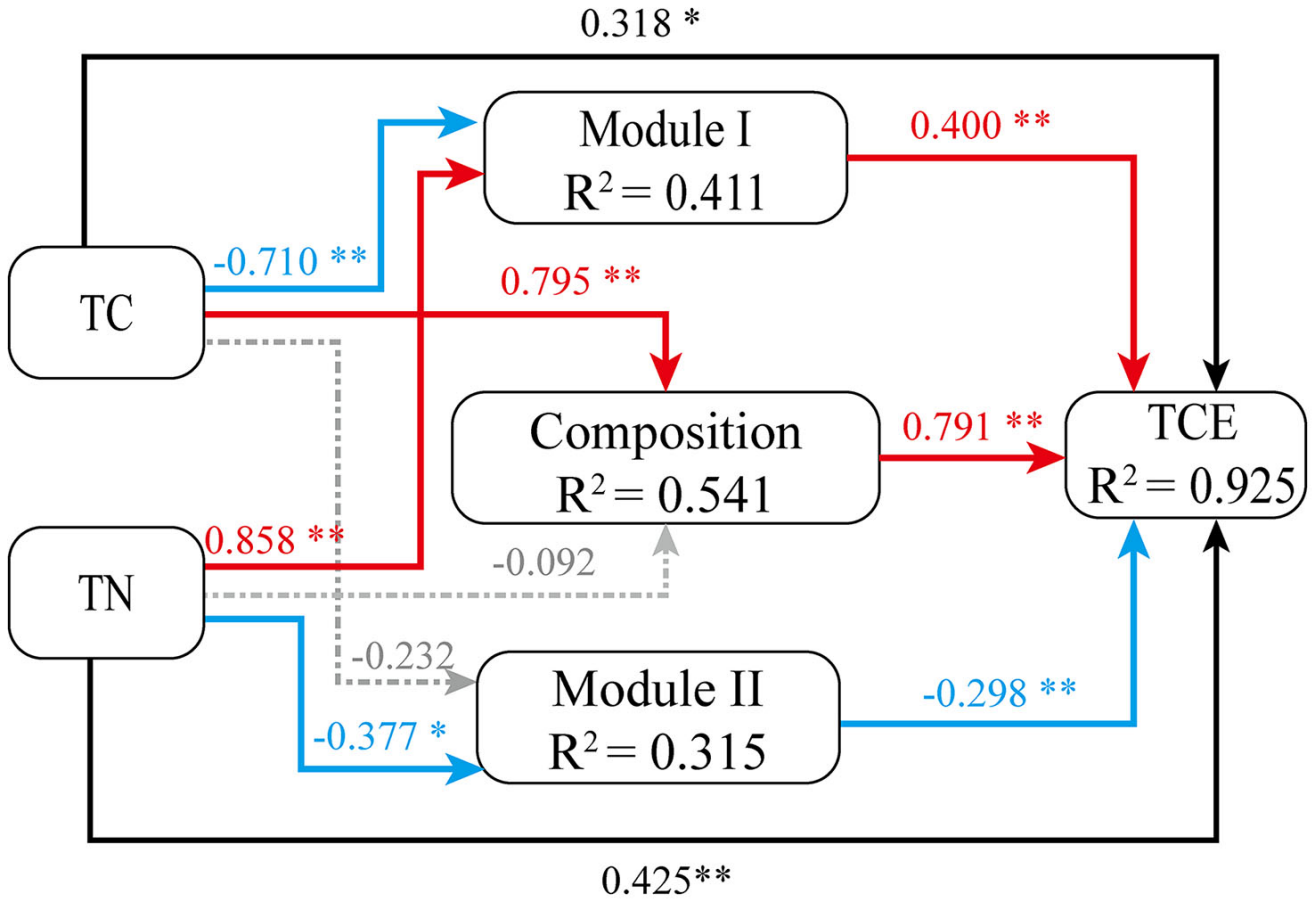
PLS path model of the associations of soil properties, bacterial community composition, and network topology on total carbon emissions. Modules I and II represent bacterial co-occurrence network modules. Numbers on arrows indicate standardized path coefficients, and values within endogenous variables indicate the explained variance. * and ** indicate significance at *p* < 0.05 and *p* < 0.01, respectively.

## 4 Discussion

### 4.1 The combined application of organic and inorganic fertilizers promotes high yield, carbon emission efficiency, and a NEP-based carbon balance

Although the measurements reported in this study were conducted in 2024 and 2025, the fertilization treatments had been continuously applied to the same plots since 2012. Thus, the observed soil and microbial responses reflect the cumulative effects of long-term fertilization rather than short-term treatment effects. Among the treatments, SC exhibited higher grain yield and carbon emission efficiency while maintaining positive NEP in both years, demonstrating superior overall performance. In contrast, although MS and SM promoted the accumulation of soil nutrients and carbon stocks, neither treatment improved the balance between crop production and respiratory carbon loss during the growing season. These findings indicate that expansion of the soil carbon pool does not necessarily result in a more favorable balance between crop-derived carbon gains and respiratory carbon losses.

Previous studies have shown that chemical fertilizers rapidly supply essential nutrients for crop growth (Xie et al., 2024), whereas organic fertilizers improve soil physicochemical properties and provide a slower and more sustained nutrient supply (Huang et al., 2024). Their combined application can better synchronize nutrient availability with crop demand, thereby enhancing productivity while mitigating carbon losses (Toth et al., 2025). By contrast, although high inputs of organic materials increase the availability of organic carbon substrates, they may also stimulate microbial respiration and consequently increase CO_2_ emissions (Ma et al., 2025; Wang et al., 2025b).

The benefits of SC extended beyond yield enhancement, as this treatment combined high grain productivity with greater carbon emission efficiency and a more favorable growing-season NEP (Wang et al., 2022b; Zhang et al., 2023). Specifically, the combination of moderate organic inputs and inorganic nutrient supply under SC may have sustained crop growth while preventing excessive respiratory carbon losses (Xie et al., 2024), thereby increasing grain yield. In contrast, although MS produced the greatest increases in TC, TN, SOC, and labile carbon fractions, its higher TCE and lower NEP suggest that the large straw input was accompanied by substantially greater respiratory CO_2_ losses. However, because the sources of respired CO_2_ were not partitioned, the observed emissions cannot be attributed exclusively to straw-derived carbon (Wang et al., 2024, 2026).

Interannual variation further supports this interpretation. Greater rainfall in 2025 than in 2024 enhanced the yield-promoting effects of SC and CF, whereas MS exhibited a more pronounced increase in carbon emissions. This pattern suggests that water availability interacts with fertilization regime to regulate crop production and respiratory carbon losses (Gao et al., 2017; Wang et al., 2025a). From both agronomic and ecological perspectives, the integrated application of organic and inorganic fertilizers may therefore represent a more stable and effective management strategy for semi-arid dryland maize systems.

### 4.2 Fertilization regimes reshape bacterial community composition with distinct functional implications for carbon utilization

Soil bacterial communities represent a critical biological link between fertilization practices and carbon cycling in cropland ecosystems (Bhattacharyya et al., 2022; Wei et al., 2024). Fertilization exerted a stronger effect on bacterial community composition than on bacterial abundance or α-diversity, consistent with previous findings from the same long-term experimental site on the semiarid Loess Plateau (Wang et al., 2022b). These results suggest that fertilization may regulate carbon turnover by selecting taxa with distinct resource-acquisition strategies, even when overall bacterial abundance and richness remain relatively stable.

The bacterial taxa enriched under MS were ecologically consistent with the substrate-rich conditions created by substantial straw inputs. MS increased the relative abundances of Proteobacteria, Bacteroidota, Gammaproteobacteria, Bacteroidia, Chitinophagaceae, and Microscillaceae (Yang et al., 2025). Previous straw-return and metagenomic studies have linked several of these groups to the rapid utilization of fresh organic substrates and the degradation of plant-residue polysaccharides (Ding et al., 2025). Their enrichment therefore indicates the development of a straw-responsive, decomposer enriched community dominated by potential decomposers under MS. By contrast, SC and CF selected for communities characterized by higher relative abundances of Alphaproteobacteria, Sphingomonadaceae, and Gemmatimonadota. Similar shifts in bacterial community composition have been reported in studies combining reduced mineral fertilizer application with organic inputs (Jin et al., 2022; Shi et al., 2023). Members of Sphingomonadaceae include rhizosphere-associated microorganisms capable of utilizing diverse plant-derived compounds, whereas Gemmatimonadota are commonly abundant in arid and semiarid soils. Their enrichment under SC and CF is thus consistent with resource environments governed primarily by root-derived carbon inputs, mineral nutrient availability, and seasonal water limitation.

Long-term fertilization may have favored these potential topological keystone ASVs by generating heterogeneous carbon substrates and nitrogen niches. Xanthomonadaceae and Sphingomonadaceae may contribute to the turnover of plant-derived compounds, whereas Xanthobacteraceae and Nitrosomonadaceae may link carbon utilization to nitrogen transformations. Gemmatimonadaceae may additionally enhance community persistence under seasonal water limitation (Banerjee et al., 2018b). These complementary ecological strategies may help coordinate carbon and nitrogen cycling and maintain bacterial network organization. However, these inferred roles require experimental validation. Moreover, PICRUSt2 predicts genomic functional potential from 16S rRNA gene data rather than directly measuring gene expression or metabolic activity (Douglas et al., 2020). Thus, the network and functional analyses collectively suggest that fertilization-induced changes in resource availability reorganized bacterial associations and functional potential, but direct measurements are needed to confirm the underlying ecological processes.

### 4.3 Soil carbon fractions and bacterial community structure jointly shape carbon sequestration-emission coupling

Compared with SOC, activity organic carbon fractions are more sensitive to fertilization. Among these fractions, POC and WSOC are important indicators of soil responses to different fertilization regimes because their dynamics more directly reflect the potential for soil carbon decomposition and utilization (Li et al., 2016). In this study, POC and WSOC responded more strongly to fertilization than did SOC and were more closely associated with TCE. Fertilization-induced changes in soil carbon composition are generally accompanied by shifts in greenhouse gas emission patterns. In particular, under the combined application under MS, the expansion of the soil carbon pool was accompanied by an increase in labile carbon fractions, potentially leading to greater respiratory carbon losses (Wang et al., 2025b). The strong relationships between TCE and both POC and WSOC further highlight the limitations of evaluating the carbon sequestration effects of fertilization solely based on total SOC. Therefore, carbon sequestration and carbon emissions should not be viewed as opposing endpoints along a single SOC gradient but rather as parallel processes jointly regulated by carbon inputs, carbon forms, carbon stability, and microbial utilization (Wang et al., 2024, 2026).

Studies conducted in the semiarid region of the Loess Plateau have shown that fertilization-induced changes in CO_2_ emissions are influenced not only by soil carbon and nitrogen conditions but also by bacterial community composition and co-occurrence network structure (Wang et al., 2022b, 2024). Consistent with these findings, TCE in the present study was more strongly associated with bacterial community composition and network structure than with bacterial abundance or diversity. Collectively, these results suggest that soil respiration is governed primarily not by bacterial abundance or species richness itself (Wang et al., 2022b), but by the selective enrichment of bacterial taxa with distinct carbon-utilization strategies in response to changes in soil carbon composition. These community-level shifts ultimately influence soil carbon decomposition and emissions (Chen et al., 2019; Nie et al., 2024).

Fertilization can regulate the balance between crop-derived carbon inputs and respiratory carbon losses by altering the availability of soil carbon and nitrogen resources and restructuring microbial communities (Wang et al., 2022b). The structural equation model developed in this study further supports this interpretation. Fertilization initially alters the quantity and forms of carbon and nutrient inputs, thereby affecting crop growth dynamics and the stable soil carbon pool. The resulting changes in resource availability and environmental conditions subsequently select for specific bacterial communities and shape their co-occurrence networks; together, these effects ultimately regulate NEP (Wang et al., 2022b). However, this study did not systematically evaluate fungal decomposers, changes in the chemical forms of carbon, seasonal succession in bacterial communities, functional gene expression, carbon fluxes during the non-growing season, or the specific sources of CO_2_ emissions. Future studies should integrate bacterial and fungal community sequencing with enzyme activity assays or transcriptomic analyses, repeated sampling across multiple seasons, characterization of the carbon properties of organic amendments, and comprehensive ecosystem carbon-budget assessments or stable-isotope analyses. Such approaches would enable more rigorous testing of the mechanisms proposed above (Wang et al., 2021; Ingwersen et al., 2024).

## 5 Conclusion

Long-term fertilization significantly affected maize yield, carbon and nitrogen accumulation, carbon emissions, and bacterial community composition in the semi-arid dryland maize system. The SC treatment maintained a positive growing-season NEP within the accounting framework used in this study and produced the highest grain yield and carbon emission efficiency. These findings demonstrate that SC effectively balanced crop carbon sequestration against respiratory carbon losses. Microbial community analysis revealed that MS enriched Proteobacteria, Bacteroidota, and related taxa, whereas SC and CF favored Alphaproteobacteria, Sphingomonadaceae, and Gemmatimonadota. Differences in carbon emissions were jointly driven by soil carbon and nitrogen pools, microbial community composition, and microbial network interactions. In areas with similar soil and climatic conditions, replacing 50% of mineral nitrogen with 3.03 t ha^−1^ of commercial organic fertilizer (equivalent to 100 kg N ha^−1^), combined with 150 kg P_2_O_5_ ha^−1^, can simultaneously enhance maize productivity and optimize the system-level carbon balance. In practice, this fertilization strategy should be adjusted according to soil fertility, precipitation patterns, and the properties of the organic fertilizer.

## Data Availability

The datasets presented in this study can be found in online repositories. The names of the repository/repositories and accession number(s) can be found at: https://www.ncbi.nlm.nih.gov/bioproject/PRJNA1524975.
