# Supplementary material for: Organic-inorganic fertilization regulates carbon sequestration-emission coupling through soil carbon fractions and bacterial community structure in dryland maize fields
Source: Front Microbiol. 2026 Sep 28;17:1901767. doi: 10.3389/fmicb.2026.1901767 (PMC13655126; doi:10.3389/fmicb.2026.1901767)
Supplement: Supplementary file 1 [file Supplementary_file_1.docx]

Supplementary files–Table S

Table S 1. Topological characteristics and taxonomic information of potential keystone ASVs in the bacterial co-occurrence network.

| ID | Phylum | Class | Family | Module | Degree | BC | CC | EC |
| --- | --- | --- | --- | --- | --- | --- | --- | --- |
| ASV52 | *Proteobacteria* | *Gammaproteobacteria* | *Xanthomonadaceae* | 1 | 66 | 780.53 | 0.31 | 0.74 |
| ASV796 | *Bacteroidota* | *Bacteroidia* | *env.OPS_17* | 1 | 64 | 547.11 | 0.31 | 0.70 |
| ASV603 | *Proteobacteria* | *Alphaproteobacteria* | *Xanthobacteraceae* | 1 | 63 | 562.08 | 0.32 | 0.75 |
| ASV82 | *Actinobacteriota* | *Actinobacteria* | *Streptomycetaceae* | 1 | 62 | 622.00 | 0.31 | 0.70 |
| ASV479 | *Gemmatimonadota* | *S0134_terrestrial_group* | *S0134_terrestrial_group* | 1 | 60 | 294.46 | 0.31 | 0.68 |
| ASV672 | *Gemmatimonadota* | *Longimicrobia* | *Longimicrobiaceae* | 1 | 58 | 340.01 | 0.30 | 0.68 |
| ASV7 | *Proteobacteria* | *Alphaproteobacteria* | *Sphingomonadaceae* | 1 | 56 | 917.16 | 0.31 | 0.61 |
| ASV590 | *Bacteroidota* | *Bacteroidia* | *Microscillaceae* | 1 | 56 | 284.19 | 0.31 | 0.66 |
| ASV134 | *Proteobacteria* | *Gammaproteobacteria* | *Xanthomonadaceae* | 1 | 55 | 394.06 | 0.31 | 0.63 |
| ASV692 | *Gemmatimonadota* | *Gemmatimonadetes* | *Gemmatimonadaceae* | 1 | 55 | 390.22 | 0.30 | 0.61 |
| ASV40 | *Gemmatimonadota* | *Gemmatimonadetes* | *Gemmatimonadaceae* | 1 | 54 | 740.44 | 0.31 | 0.59 |
| ASV751 | *Bacteroidota* | *Bacteroidia* | *Chitinophagaceae* | 1 | 54 | 484.18 | 0.30 | 0.60 |
| ASV5 | *Gemmatimonadota* | *Gemmatimonadetes* | *Gemmatimonadaceae* | 1 | 53 | 583.15 | 0.30 | 0.54 |
| ASV1428 | *Gemmatimonadota* | *Longimicrobia* | *Longimicrobiaceae* | 1 | 53 | 241.29 | 0.30 | 0.63 |
| ASV265 | *Proteobacteria* | *Gammaproteobacteria* | *Nitrosomonadaceae* | 1 | 51 | 317.37 | 0.30 | 0.61 |
| ASV57 | *Proteobacteria* | *Gammaproteobacteria* | *Nitrosomonadaceae* | 1 | 50 | 589.69 | 0.30 | 0.47 |
| ASV248 | *Gemmatimonadota* | *BD2-11_terrestrial_group* | *BD2-11_terrestrial_group* | 2 | 48 | 756.42 | 0.31 | 0.55 |
| ASV178 | *Proteobacteria* | *Gammaproteobacteria* | *Comamonadaceae* | 2 | 44 | 382.13 | 0.31 | 0.44 |
| ASV319 | *Actinobacteriota* | *Acidimicrobiia* | *uncultured* | 1 | 44 | 459.79 | 0.31 | 0.49 |
| ASV605 | *Proteobacteria* | *Alphaproteobacteria* | *uncultured* | 1 | 43 | 176.51 | 0.30 | 0.52 |

**Degree represents node connectivity; CC refers to closeness centrality; BC refers to betweenness centrality; and EC indicates eigenvector centrality.**

Supplementary files–Figure S

Figure S 1. Rainfall for 2024, 2025, and multi-year average.

Figure S 2. Schematic diagram of the full-film double-row furrow planting system and aerial photo of the experimental field

Figure S 3. Network positions of the 20 potential topological keystone ASVs in the bacterial co-occurrence network. The left panel shows the complete bacterial co-occurrence network, and the area enclosed by the red rectangle is enlarged in the right panel. Potential topological keystone ASVs are highlighted as cyan nodes and labeled with their ASV identifiers, whereas the remaining ASVs are shown as gray nodes. Node size is proportional to degree. Magenta and cyan edges represent significant positive and negative correlations, respectively. The red arrow indicates the magnified network region.

Figure S 4. Predicted KEGG Level 1 and Level 2 functional profiles of bacterial communities under different fertilization regimes.

Figure S 5. Regression relationships between the relative abundances of dominant bacterial phyla and soil organic carbon, total carbon, total nitrogen, and carbon emissions. (A-D) Proteobacteria; (E-H) Actinobacteriota; and (I-L) Gemmatimonadota. SOC, TC, TN, and CE denote soil organic carbon, soil total carbon, soil total nitrogen, and carbon emissions, respectively.

Figure S 6. Regression relationships between the relative abundances of dominant bacterial classes and soil organic carbon, total carbon, total nitrogen, and carbon emissions. (A-D) Gammaproteobacteria; (E-H) Alphaproteobacteria; and (I-L) Gemmatimonadetes. SOC, TC, TN, and CE denote soil organic carbon, soil total carbon, soil total nitrogen, and carbon emissions, respectively.

Figure S 7. Regression relationships between the relative abundances of dominant bacterial families and soil organic carbon, total carbon, total nitrogen, and carbon emissions. (A-D) Sphingomonadaceae; (E-H) Gemmatimonadaceae; and (I-L) Nitrosomonadaceae. SOC, TC, TN, and CE denote soil organic carbon, soil total carbon, soil total nitrogen, and carbon emissions, respectively.
